# ​The Reconstruction of a Massive Occipital Defect With a Bilateral Temporal Advancement Flap Following Advanced Melanoma Resection: A Case Report

**DOI:** 10.7759/cureus.107455

**Published:** 2026-04-21

**Authors:** José D Rodríguez Enríqeuz, Enrique Córdova López, Miguel A Bautista Badal, Paul A Palacios Zaragoza, Azucena D Bernal González

**Affiliations:** 1 General Surgery, Hospital Regional de Alta Especialidad Gustavo A Rovirosa, Villahermosa, MEX; 2 Medicine, Universidad Olmeca, Villahermosa, MEX; 3 Medical Physics, Universidad de Colima, Colima, MEX; 4 General Practice, Universidad Autónoma del Estado De México, Toluca, MEX

**Keywords:** melanoma surgery, occipital area, scalp reconstruction, subgaleal space, temporal myofascial flap

## Abstract

Scalp reconstruction following oncological resection is particularly challenging due to limited tissue laxity and the convexity of the region. Large occipital defects must be carefully reconstructed to provide adequate coverage and minimize morbidity. We present the case of a 71-year-old man with stage IV advanced occipital melanoma (Breslow 7 mm, Clark level IV), in which the melanoma was locally excised over a 15 × 15 cm area, resulting in a large defect. A bilateral temporal advancement flap was employed for reconstruction, based on the region of relative laxity and within the subgaleal avascular plane, while the supraorbital and supratrochlear neurovascular bundles were preserved. The patient showed a good postoperative course. This report demonstrates the validity of the bilateral temporal advancement flap as a robust, reliable, and aesthetically satisfactory option for the treatment of large posterior scalp defects, thereby eliminating the need for skin grafting or free tissue transfer.

## Introduction

The scalp is a frequent site for cutaneous malignancies, including melanoma, which often requires wide local excision to achieve negative margins [1]. Reconstruction is challenging due to the scalp’s limited elasticity, convex bony structure, and hair-bearing skin [2]. While primary closure is suitable for small defects (typically those less than 3 cm), larger defects require more complex reconstructive approaches and alternative tissue transfer techniques [3].

Partial or full-thickness skin grafts may be an option, although they carry a margin of error that may result in aesthetic failure, graft loss, alopecia, and low overall durability, especially when there is bone exposure, which constitutes a contraindication to grafting this type of defect, or when secondary postoperative radiotherapy is required [4,5]. On the other hand, free flaps provide reliable coverage; however, not all hospitals or facilities have microsurgical equipment and trained personnel to perform this technique, nor do they always ensure adequate follow-up to reduce the incidence of complications associated with these flaps, which can lead to flap failure and increased morbidity. Hence, local rearrangement of adjacent tissue (rotation, transposition, or advancement flaps) remains a viable option for repairing these types of defects, provided it is performed with proper planning and technique [5,6].

However, one of the surgical challenges of flap advancement or rotation is the limited elasticity and quantity of tissue available to cover a given defect, which can lead to failure due to poor planning. In these cases, the bilateral temporal advancement flap can effectively mobilize tissue by taking advantage of the greater elasticity and lower rigidity of the temporal and frontal regions [7,8]. Therefore, it is a reliable reconstructive option for complex occipital defects. This case report presents the feasibility, surgical technique, and clinical results of using a bilateral temporal advancement flap for the reconstruction of a 15 × 15 cm occipital scalp defect following resection of an advanced-stage IV melanoma, with a favorable clinical course, representing a successful approach in the surgical management of one of the most common cancers worldwide.

## Case presentation

**Figure 1:**
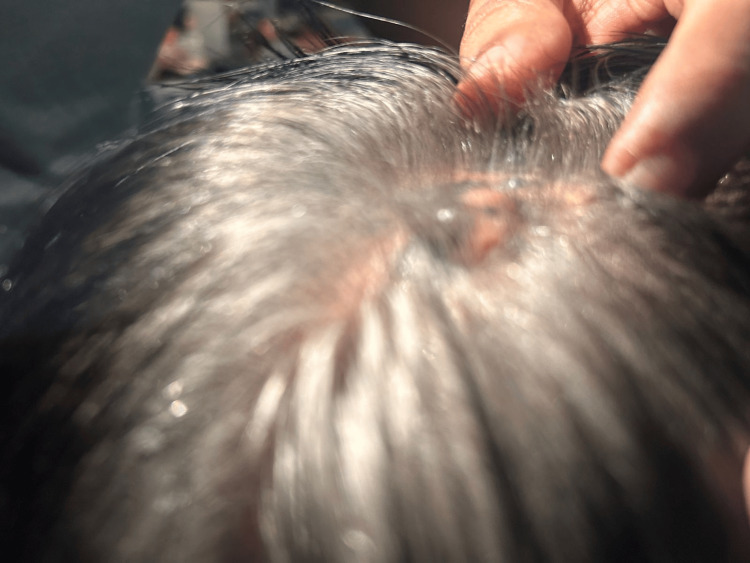
Preoperative assessment - image 1 Clinical presentation of the occipital lesion. A 5 x 3 cm hyperpigmented, multi-lobulated, and non-ulcerated tumor is observed in the central parieto-occipital region. Two satellite lesions are identified within a 1 cm radius of the primary tumor, consistent with the diagnosis of advanced malignant melanoma

**Figure 2:**
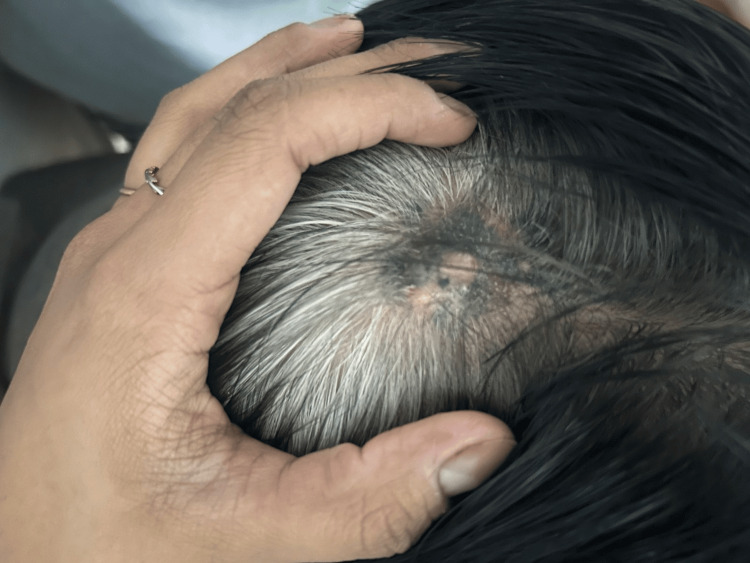
Preoperative assessment - image 2 Clinical presentation of the occipital lesion. A 5 x 3 cm hyperpigmented, multi-lobulated, and non-ulcerated tumor is observed in the central parieto-occipital region. Two satellite lesions are identified within a 1 cm radius of the primary tumor, consistent with the diagnosis of advanced malignant melanoma

The patient was a 71-year-old male whose current illness had begun with a hyperpigmented lesion, with irregular borders and rapid growth, on the scalp of the occipital region, which had been present for one year. His medical history included the following significant findings: type 2 diabetes mellitus and systemic hypertension, both controlled with medication. Physical examination revealed an asymptomatic, non-ulcerated hyperpigmented lesion, approximately 5 x 3 cm, with two adjacent satellite lesions (Figures 1, 2).

The patient's diagnosis of malignant melanoma was confirmed preoperatively by biopsy. Subsequent staging of the disease was performed using a contrast-enhanced thoracoabdominopelvic CT scan, which revealed bilateral basal pulmonary nodules and a metastatic lesion in liver segment VIII, confirming clinical stage IV. Due to the presence of systemic metastases, a wide local excision was strictly indicated for palliative control to prevent tumor ulceration, bleeding, lesion progression, and increased morbidity. Several factors were considered in the surgical planning of the approach and defect reconstruction, including surgical morbidity and expected survival based on the disease stage. A bilateral, single-stage, temporary advancement flap was chosen. This approach provided rapid and reliable wound coverage and minimized physiological burden, avoiding the prolonged surgical time and donor-site morbidity associated with free tissue transfer and the availability of microsurgical equipment. All preoperative laboratory results were within normal limits.

**Figure 3:**
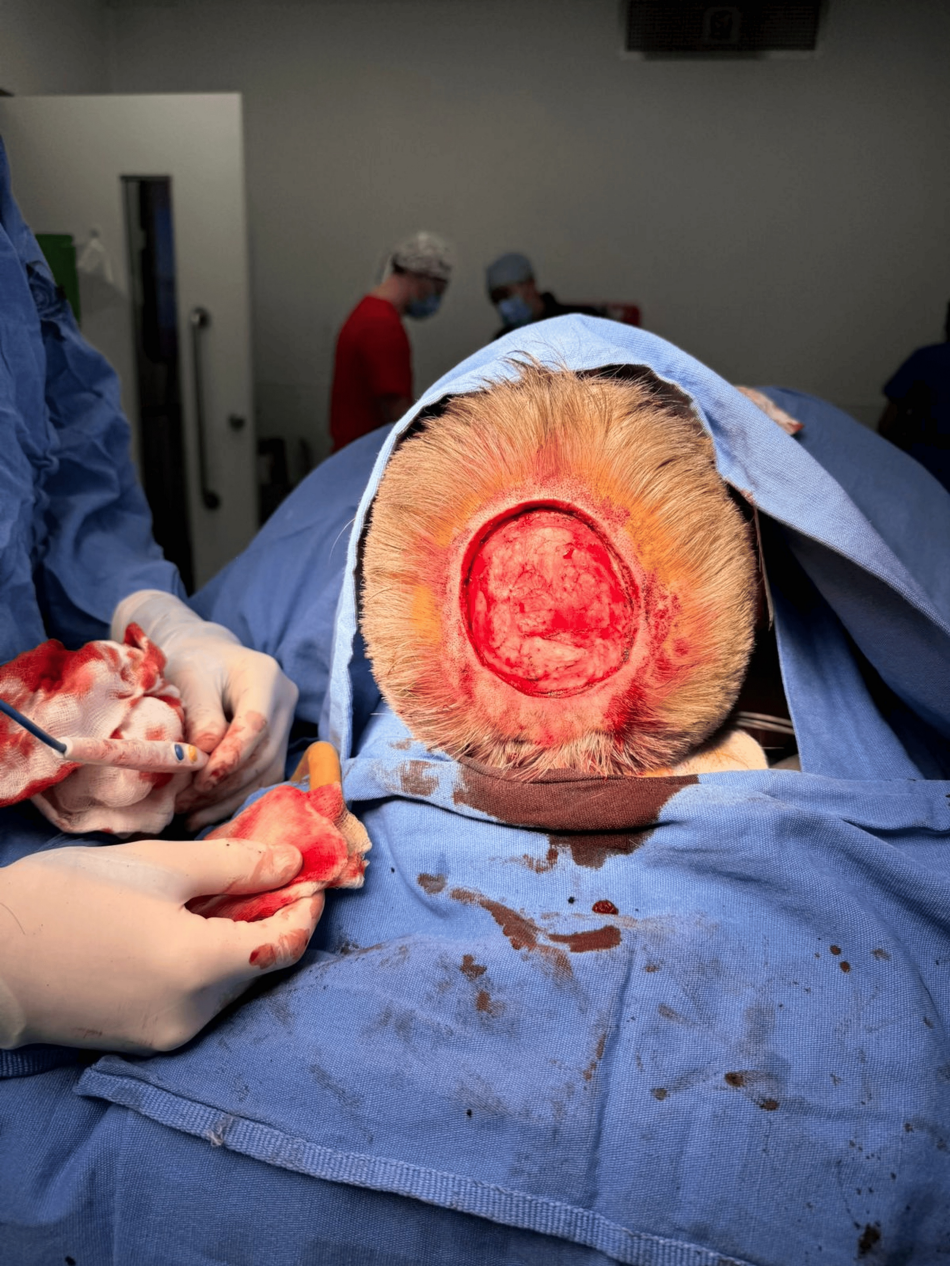
Surgical defect Wide local excision and resulting defect. Intraoperative view following wide oncological resection with macroscopic margins greater than 2 cm. The procedure resulted in a massive 15 x 15 cm scalp defect with exposure of the underlying calvarium (periosteum preserved). This defect size highlights the limited availability of local tissue for primary closure

In the operating room, the patient was placed in the supine position under general anesthesia. The area was prepared aseptically and antiseptically, and sterile drapes were applied. A wide resection of the multilobulated, vegetating tumor (4 x 3 cm) and associated satellite lesions was performed, ensuring macroscopic margins greater than 2 cm. This resulted in a massive 15 x 15 cm occipital defect with exposure of the underlying skull (Figure 3).

**Figure 4:**
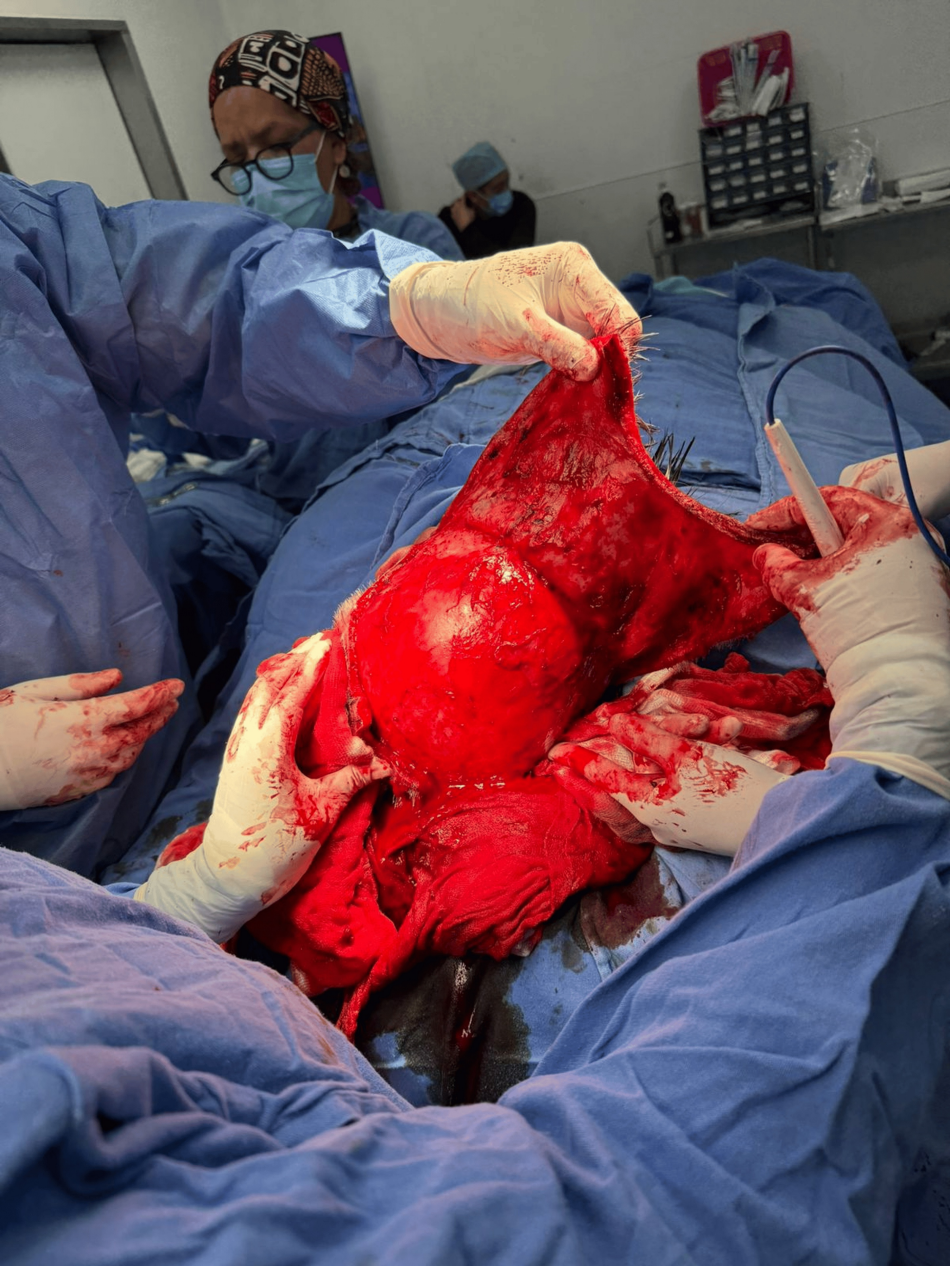
Flap design and dissection ​Posterior mobilization of the bipedicled visor flap. The elevated scalp tissue is advanced posteriorly to bridge the 15 x 15 cm occipital gap. The relative laxity of the temporal and frontal regions allows for tension-free coverage of the posterior defect without the need for additional rotation or skin grafting

To reconstruct the defect, a bilateral temporal advancement flap was designed. Linear preauricular incisions were made bilaterally, approximately 1 mm from the tragus. Dissection proceeded anteriorly and superiorly, strictly respecting the avascular subgaleal plane (Figure 4).

**Figure 5:**
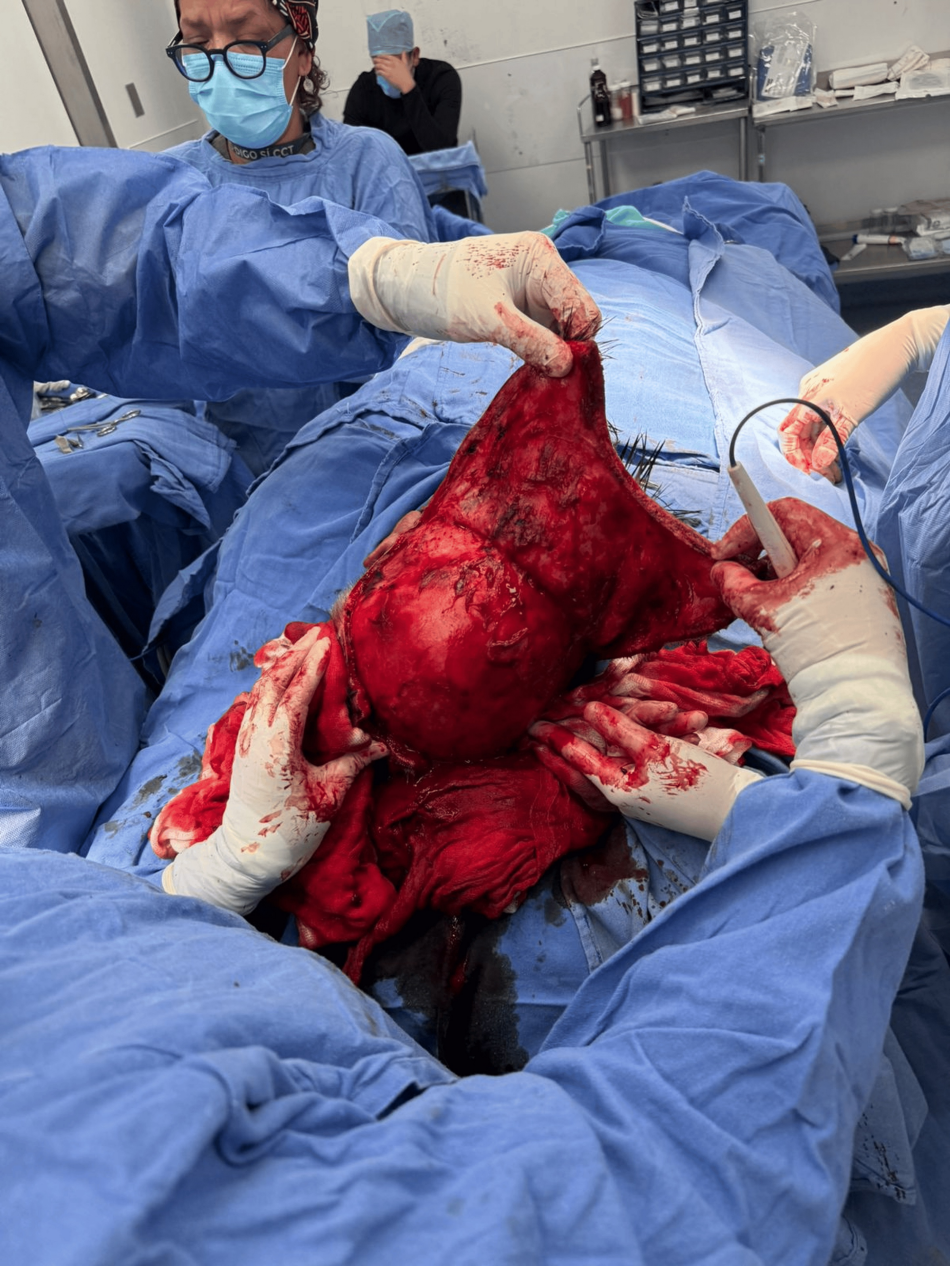
Flap advancement ​Posterior mobilization of the bipedicled visor flap. The elevated scalp tissue is advanced posteriorly to bridge the 15 x 15 cm occipital gap. The relative laxity of the temporal and frontal regions allows for tension-free coverage of the posterior defect without the need for additional rotation or skin grafting

Extensive undermining was carried out anteriorly up to the supraorbital rims and laterally towards the temporal fossae to maximize flap mobility. Upon identifying the frontal crest, the dissection plane was superficialized to preserve the insertion of the eyebrows and the origins of the supratrochlear and supraorbital arteries. Intraoperative assessment of these neurovascular bundles confirmed robust arterial pulsation, ensuring reliable vascularity for the bipedicled flap. During posterior advancement, closure tension was continuously evaluated; the wide subgaleal release significantly reduced resistance, enabling a secure, tension-free closure over the massive occipital defect (Figure 5).

**Figure 6:**
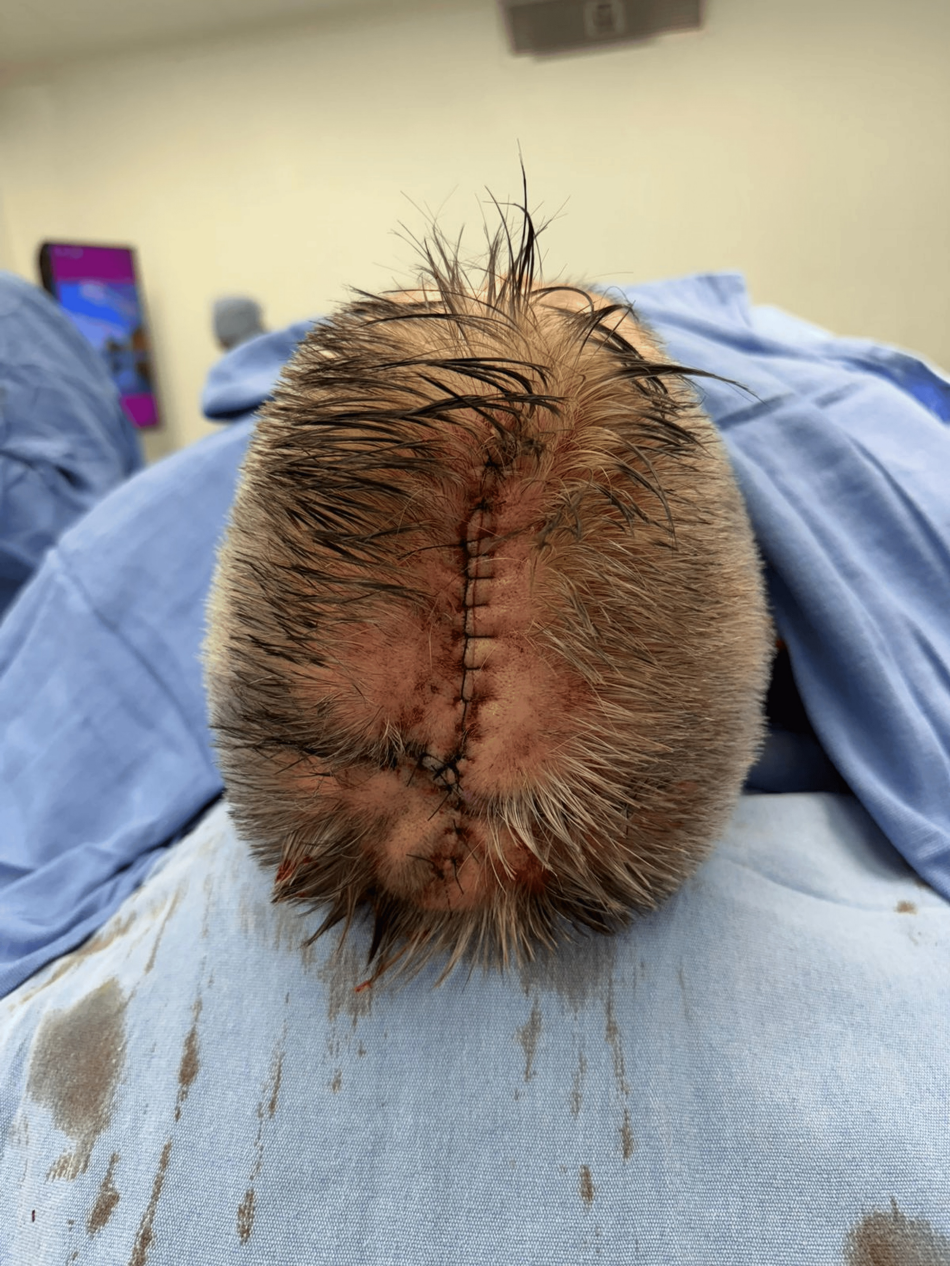
Final closure ​Immediate postoperative result. Final appearance after suturing with 2-0 non-absorbable material. Bilateral preauricular incisions are closed, and two closed-suction drains (1/4-inch Drenovac) are placed with exits via the preauricular sites to prevent hematoma formation. The reconstructed area shows adequate contour and restoration of the posterior hairline

Before complete closure, anchoring sutures were placed in all surgical wounds using 2-0 non-absorbable material. Two 1/4-inch closed-suction drains (Drenovac) were placed bilaterally with preauricular exits to prevent hematoma formation. Drain function was corroborated, and the surgical site was dressed with sterile gauze and specialized dressings (Figure 6).

Histopathological review of the surgical specimen confirmed a malignant melanoma, Breslow depth of 7 mm, Clark level IV, with 6 mitoses per high-power field, present lymphocytosis, and tumor-free margins.

**Figure 7:**
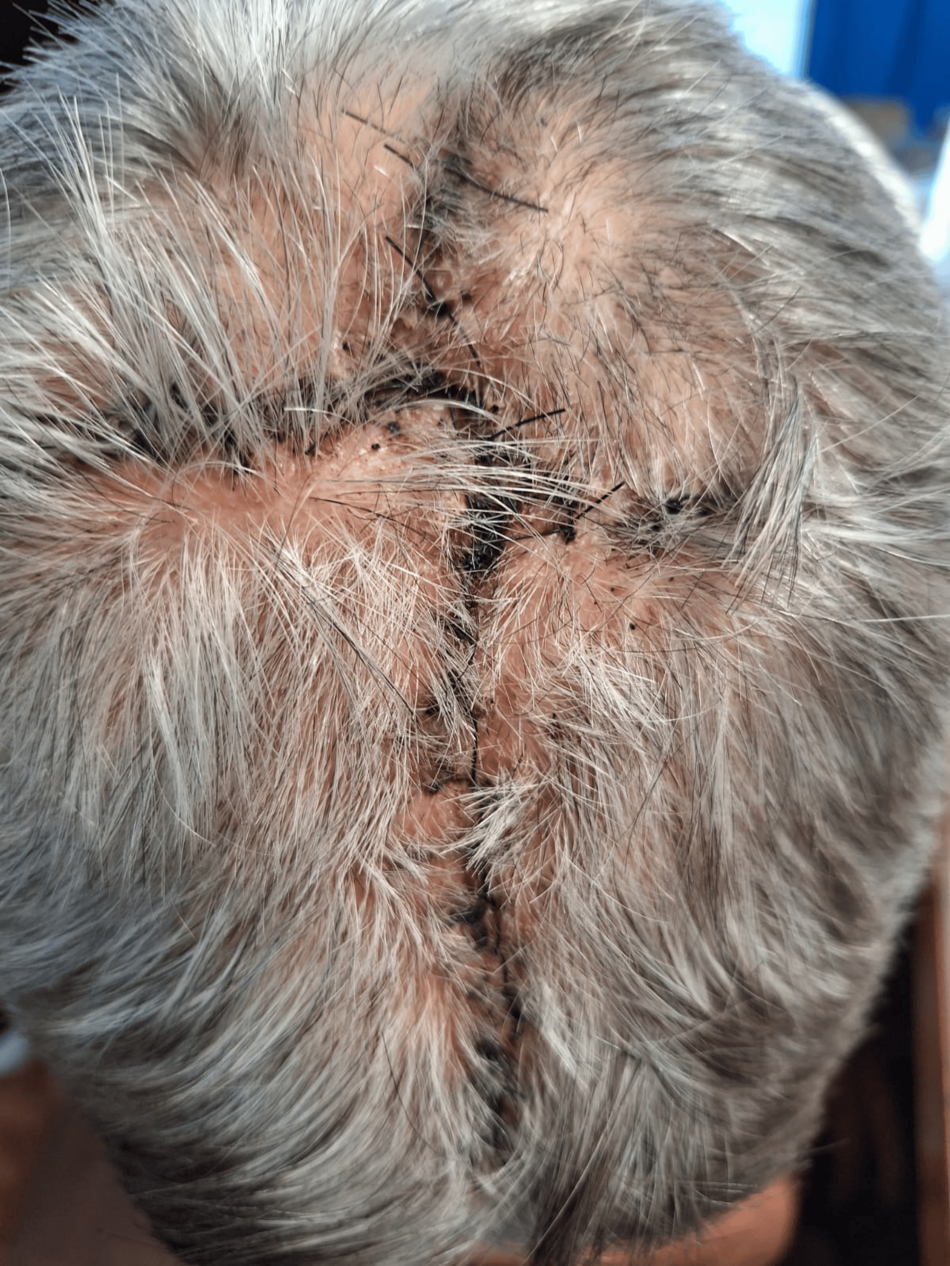
Postoperative follow-up at 15 days Clinical appearance of the reconstructed scalp 15 days after the surgery. The bilateral temporal advancement flap demonstrates 100% viability with complete absence of marginal necrosis, infection, or wound dehiscence. Adequate tissue healing, proper wound apposition, and early hair regrowth are observed along the suture lines, confirming the robust vascular supply preserved during the subgaleal dissection

At the short-term postoperative follow-up (15 days), the flap demonstrated 100% viability. The patient's recovery was unremarkable, with no postoperative complications such as hematoma, seroma, surgical site infection, or flap necrosis (Figure 7). The surgical site healed adequately, and the patient reported relief from local symptoms, successfully achieving the palliative goals. Following a rapid and uncomplicated surgical recovery, the patient was referred to the medical oncology department to continue systemic therapy management for his stage IV metastatic disease.

## Discussion

Reconstructing massive occipital defects, such as the 15 x 15 cm area in our case, presents a significant surgical challenge due to the scalp's convexity and intrinsic tissue inelasticity [1]. The primary reconstructive goals involve achieving durable calvarial coverage, promoting rapid wound healing, and maintaining an acceptable aesthetic contour, including hairline preservation [2].

Rationale for adopting the technique

In the context of a palliative resection for an advanced stage IV melanoma, the choice of reconstruction is critical. The surgical rationale must strictly balance the need for immediate, reliable local control against the imperative to minimize surgical morbidity [1,7]. For our patient, the objective was to prevent imminent complications like tumor ulceration and bleeding without subjecting him to a highly demanding physiological burden.

Review of the current literature

Various techniques are described in the literature for scalp reconstruction. While split-thickness or full-thickness skin grafts offer a simpler solution, they are frequently associated with poor durability, alopecia, and shear instability over the periosteum, making them suboptimal for large, deep occipital defects [3,4,7]. Conversely, free tissue transfer provides excellent and robust coverage. However, free flaps require prolonged operative times, microsurgical expertise, and carry significant donor-site morbidity [7,9]. Given our patient’s systemic disease burden, free tissue transfer was considered an unfavorable and overly aggressive option.

Advantages and limitations

The bilateral temporal advancement flap serves as an optimal middle ground. Its primary advantage lies in successfully recruiting tissue from the relatively lax temporal and frontal regions [6,10]. By executing bilateral preauricular incisions and dissecting strictly within the avascular subgaleal plane, the entire scalp vertex can be mobilized as a bipedicled visor flap and advanced posteriorly. This technique allows for safe, rapid dissection while preserving the critical superficial temporal, supraorbital, and supratrochlear vascular pedicles. Furthermore, it provides a "like-for-like" tissue match, yielding superior aesthetic and functional outcomes compared to skin grafting [4,6]. The main limitation of this technique is the finite amount of advancement possible; overly aggressive traction can lead to marginal necrosis or undesirable distortion of the anterior hairline. In our procedure, this was carefully mitigated by superficializing the dissection plane near the frontal crest to release tension safely.

Clinical implications

The successful application of this technique underscores its immense clinical value for large posterior defects. It provided the patient with immediate, robust coverage in a single surgical stage, eliminating the need for secondary procedures. Careful management of potential dead space, as achieved with closed-suction drains, is critical to prevent hematoma and seroma formation, which are the primary threats to flap viability in these extensive dissections.

While this case provides valuable technical insights, we acknowledge the inherent limitations of a single case report. The absence of long-term follow-up and objective cosmetic scoring (e.g., alopecia assessment or patient satisfaction scales) represents a limitation, which is primarily attributed to the patient's advanced stage IV disease and the strict palliative intent of the procedure.

## Conclusions

This report demonstrates the feasibility and reliability of the bilateral temporal advancement flap for the reconstruction of large occipital defects. It provides robust, well-vascularized tissue coverage with acceptable short-term cosmetic results and minimal donor-site morbidity. While a single case report cannot establish superiority over other well-established techniques, this local flap represents a viable and pragmatic option in the reconstructive armamentarium. It is particularly valuable in palliative settings or advanced disease stages, where rapid wound closure, reliable local control, and minimization of the physiological surgical burden are paramount.
